# Effect of partial medial meniscectomy on anterior tibial translation in stable knees: a prospective controlled study on 32 patients

**DOI:** 10.1186/2052-1847-5-17

**Published:** 2013-08-30

**Authors:** Kaissar Yammine

**Affiliations:** 1 Department of Orthopedics and Sports Medicine, The Center for Evidence-Based Sports and Orthopedic Research, Emirates Hospital, Jumeirah Beach Road, P.O.Box. 73663, Dubai, United Arab Emirates

## Abstract

**Background:**

Quantitative measurement of anterior translation of the tibia (ATT) by KT 1000 is used mainly to provide an objective assessment of knee laxity after anterior cruciate ligament (ACL) tears or ACL reconstructions. Only few papers described its use after menisectomies in knees with intact ACL. The objective of this paper is to determine whether partial medial meniscectomies could induce significant immediate post-operative ATT.

**Methods:**

Thirty-two patients with a diagnosis of partial medial meniscal tear limited to the posterior horn and documented with magnetic resonance imaging (MRI) were assessed under anesthesia before and immediately after arthroscopic meniscectomy. The assessment was performed by the same examiner by means of the MEDmetric(R) KT-1000 instrument using manual maximum (MM) force. The opposite knees were also assessed.

**Results:**

There is a significant difference between pre and post-operative KT MM mean values for the operated knees (CI: -3.933953 to −2.947297, p < 0.0001). No significance was found between the mean values for the contralateral knees before and after the completion of the menisectomy on the operated knees (p = 0.4). For the operated knees, 14 (43.75%) had a side-to-side difference between pre-and post-operative values of more than 3 mm, whereas for the contralateral knees, only 2 (6%) had the same.

**Conclusion:**

Less than half of operated knees showed significant side-to-side difference values of ATT (>3 mm), immediately after meniscectomies in unconscious patients. Our values might reflect a temporarily increase of anterior laxity under specific conditions but whether a significant laxity remains in some knees, such changes may lead to higher cartilage loading and early osteoarthritis.

## Background

Knee stability is provided by a complex interaction of a multitude of factors, including cruciate and collateral ligaments, other soft-tissue restraints, condylar geometry, tibio-femoral loading forces at the joint interfaces and active muscular control and balance.

The primary restraint for the ATT is the ACL and in particular the antero-medial bundle which, when it is cut, induces a significant translation of the tibia [1-5]. Other structures, called secondary restraints, have little or no impact on ATT when injured; lesions of the anterolateral femoro-tibial ligament, the medial collateral ligament,or the lateral capsular ligament could increase the ATT only when the ACL is ruptured [4,5]. Vertical compressive loads on the knee joint might also mildly contribute to the anterior knee stability [6-10].

Studies showed that, during knee flexion, the medial meniscus has less excursion compared to the lateral meniscus and in particular at the level of the posterior horn [11,12]. In ACL-deficient knees, total lateral meniscectomy does not increase, in vivo, the ATT [13]. On the other hand, in vivo total excision of the medial meniscus associated to the section of the ACL allowed significantly greater increases in anterior displacement than those already increased by isolated section of the ACL [14]. A total medial meniscectomy in ACL-deficient knees seems to change the repartition of charges on the tibial surface which might induce a translation [15-17]. Most of the clinical research exploring knee stability by the KT-1000 arthrometer was related to ACL injuries, with or without meniscectomy or meniscal repair, or to the residual laxity after ACL reconstructions. The effect of partial medial meniscectomies on ATT in ACL-intact knees in the few published studies has being controversial [18-28] but there was an agreement that the medial meniscus is to be considered as a secondary restraint to anterior laxity. Most of these studies were conducted either on cadavers or on patients fully awake.

The objective of this study is to evaluate objectively the impact of partial medial meniscectomies, confined to the posterior segment, on the anterior translation of the tibia in the immediate postoperative phase while patients are under general anesthesia. We hypothesized that under such conditions medial meniscectomies would significantly increase anterior laxity when compared to normal knees.

## Methods

Between October 2010 and January 2012, forty patients engaged in sport activities and having an isolated medial meniscal tear were enrolled. The study has been approved by the Committee of Medical Ethics at our institution and a written informed consent has been obtained from all included patients. Thirty-two patients with a tear limited to the posterior horn of the medial meniscus were included in this prospective study. Eight patients were excluded during the study; after the completion of the pre-operative anesthesia consultation, six patients changed their mind and opted for a spinal anesthesia, and two other experienced recent pain in the contralateral knee. The cohort consisted of 22 males and 10 females with a median age of 28.5 (range between 20 and 44 years). All patients had their meniscal injuries documented with MRI. At the first consultation, the patients participated in the following sports: pivot-contact 10 patients (31.2%), pivot without contact 15 patients (46.8%), weight-bearing without contact 5 patients (15.6%), weight-bearing without pivot 2 patients (6.2%),

The inclusion criteria were; a) a fresh trauma of the knee sustained during sport activities; b) the injury was believed to occur at less than 3 months and at more than one month from the date of treatment; c) an isolated injury of the posterior horn of the medial meniscus; d) arthroscopy conducted under general anesthesia.

The exclusion criteria were; a) any other injury, including injuries to ACL, found on MRI or during arthroscopy; b) the presence of a degenerative knee; c) a recent trauma (< 3 months) or a history of meniscal/ligament injury to the contralateral knee; d) a symptomatic contralateral knee: flessum or effusion; e) arthroscopy conducted under spinal anesthesia.

The amount of excision was visually evaluated by the author to be between 75 and 100% of the posterior horn; in only four patients the amount of excision was between 25 and 50%.” The posterior rim was conserved in all cases and tested arthroscopically with a hook. Meniscectomy was performed using punches only. Tourniquet was applied before the surgery and deflated after taking the measurements.

### KT-1000 measurement protocol

A KT-1000 arthrometer (MEDmetric Corp., San Diego, CA) was used for the instrumental measurements of laxity (arthrometry). Each measurement was performed with the patient in the supine position, with the knee at 20° using the supplied thigh support and in neutral position. The instrument was calibrated to zero before every test. A single experienced examiner (KY) did the measurements.

The operated knee was voided from the irrigating liquid before skin suture. Adhesive dressings were applied, measurements were taken, and only after the tourniquet was released. The rationale behind this sequence was our intention to perform measurements on operated knees under, as much as possible, the same conditions.

Four sets of measurements were conducted. The first consisted of measuring the ATT of the injured knee before the arthroscopy but after 5 minutes of anesthesia induction. The second measurement was done on the contralateral non-injured knee. The third one consisted of measuring the ATT on the operated knee at the end of the arthroscopy after the application of small adhesive dressings. The last was conducted on the non-operated knee at the end of the procedure. Tibial translation was measured after applying a maximal manual (MM) maneuver. Each measurement was taken three times on both sides during the same session and the average value was retained for analysis. We chose to define side-to-side difference of ≥3 mm in the anterior laxity measurements as indicating a significant difference.

### Statistical analysis

The StatsDirect statistical software (Version 2.7.8; 11/08/2011, Altrincham, UK) was used for the statistical analysis.

Unpaired student t-test was used to compare means and Chi-square test to compare the rates where translation is considered significant (> 3 mm). We defined a p-value of less than 0.05 as statistically significant.

## Results

The number of operated knees was approximately the same for each side with 18/32 (56.2%) right knees, and 14/32 (43.8%) left knees. Three types of meniscal tear were found during arthroscopy; flap with anterior pedicle: 15/32 (46.8%), horizontal tears: 10/32 (31.3%), and radial tears: 7/32 (21.8%). The amount of excision was visually appreciated by the author to be between 1 and 1.5 cm2; in only four patients the amount of excision has been evaluated to be less than 1 cm2.

The mean values and their standard deviations (SDs) of MM pre- and post-operative KT measurements for both sides are given in Table 1; pre-operative KT mean values were 8.8 mm (± 0.87) for operated knees and 8.0 mm (± 0.55) for contralateral knees, and the post-operative KT mean values were 12.25 mm (± 1.08) for operated knees and 9.0 mm (± 0.61) for contralateral knees.

**Table 1:** Mean values and SDs of MM KT measurements

	**Operated knees**		**Contralateral knees**	
	**Means (mm)**	**SD**	**Means (mm)**	**SD**
Pre-operative KT mean value	8.8	± 0.87	8.0	± 0.55
Post-operative KT mean value	12.25	± 1.08	9.0	± 0.61

There is a significant difference between pre and post-operative KT MM mean values for the operated knees (CI: -3.933953 to −2.947297, p < 0.0001). No significance was found between the mean values for the contralateral knees before and after the completion of the meniscectomy on the operated knees (p = 0.4).

For the operated knees, 14 (43.75%) had a side-to-side difference between pre-and post-operative values of more than 3 mm, whereas for the contralateral knees, only 2 (6%) had > 3 mm difference in values before and after the completion of the meniscectomyon the operated knees (Yates-corrected Chi^2^ = 10.083, p = 0.0015; OR = 11.66, CI: 2.3728 to 57.363083, p = 0.001). No statistical difference was found in relation to the type of meniscal tear or gender.

## Discussion

Our main findings consisted of a high rate of side-to-side difference values immediately after meniscectomies in unconscious patients. The effect of a partial meniscal excision on knee laxity is not well researched and this is probably due to the fact that medial menisci are considered as secondary refrains to the ATT with little effect on anterior laxity. Most published papers investigated the long-term effect of meniscectomies on the stability of the knee with either subjective or objective tests. To our knowledge this is the first study to explore the immediate impact of medial meniscectomies on ATT under anesthesia.

Long term results of some “subjective” clinical studies [18-20] showed an increase, using the anterior drawer test for assessment, in ATT after a total meniscectomy by arthrotomy in ACL-intact knees with a range between 14-36%. Hede and Sandberg [21] found no significant difference in clinical laxity rate (28.3%) after partial or total meniscectomy by arthrotomy and long term positive Lachman was found in only 6% of patients by Neyret et al. [22]. Only one study found no long-term anterior drawer sign after medial meniscectomy [23].

On the other hand, the long term results of some “objective” clinical studies using different clinical or radiological devices for laxity assessment showed non-significant increase in ATT after partial or total meniscetomies by arthrotomy [24-26]. However, one study using objective radiological assessment reported a significant increase in ATT in patients who underwent medial meniscectomy compared to those who had lateral menisectomies and to healthy knees [29]. Rillardon and Djian [27] using the KT-1000 instrument after partial arthroscopic meniscectomies to assess long-term laxity, found a 12% rate of significant increase in ATT compared to a population with normal knees, in either lateral or medial meniscectomies. All the knees where ATT was increased had their posterior segment excised. The authors did not correlate their findings with the presence or absence of osteoarthritis, a frequent long-term complication after meniscectomies, which could interfere with their laxity results.

Most cadaveric studies, using devices to assess anterior laxity, consistently showed an increase of the ATT after medial meniscectomies in ACL-deficient knees; such increase was not found for lateral meniscetomies [8,13,14]. However, a recent cadaveric study by Arno et al. [30] demonstrated that while a resection of 22% of the posterior horn of the medial meniscus did not induce a change in the antero-posterior position, a 46% resection and a 100% removal of the posterior horn resulted in a statistically significant increase of the ATT when compared to normal cadaveric knees. Those findings are in agreement with our immediate laxity status results; most of our medial meniscectmies (87.5%) had at least 75% of the posterior horn removed.

In line with Wroble et al.’s [31] suggestions on the usefulness of calculating side-to-side differences rather than absolute measurements in an attempt to reduce bias generated from the patients’ level of relaxation, we calculated both the absolute and the side-to-side differences under general anesthesia. In that, we hoped to eliminate the muscular tension factor encountered whenever patients are fully awake. Regarding absolute values, our findings on the mean anterior displacement in normal knees of 8.5 mm are in accordance with those of Daniel et al. [32,33] who reported a value of 8.4 mm. However, the distribution of our data in normal knees is different; our SD values are narrower and this could be related to our study’s subgroup of sport active population and possibly to the fact that our patients were unconscious where muscular control was absent. With regard to side-to-side difference values, we found an average of 3.45 mm in operated knees; a value less than the long-term 4.7 mm recorded by Rangger et al. [34].

Our findings showed also a rate of 43.75% of significant translation at the end of the surgery. This high rate could not be explained totally by the use of general anesthesia; such translation was found only in 2% of the contralateral non-operated knees. In agreement with our results with regard to healthy knees, Highgenboten et al. [35] demonstrated that KT-1000 measurements in the unconscious state were significantly higher than the values obtained in the conscious state in ACL-intact knees (and ACL-deficient knees as well). More, they demonstrated that under anesthesia, an increasing application of force produced increasing levels of anterior laxity whatever is the status of the ACL and in particular for ACL-deficient knees. Such results could explain, at least in part, our high rates of ATT (absolute and side-to-side difference) values. On the contrary, Sernert et al. [36] found that KT measurements in healthy knees are not different between patients under anesthesia and fully awake patients. Whether values under anesthesia are closer to the real laxity status of the studied knees remain unknown. However, we believe that, in order to have better measurement accuracy, general rather than spinal anesthesia could be helpful in conferring full muscle relaxation evenly for the two studied inferior limbs.

This study included patients with similar baseline characteristics such as the occurrence of knee injuries during sport activities, meniscal tears conferred only to the posterior horn, and the use of same type of anesthesia. However, some limitations need to be discussed. *First*, young male were predominant in our study due to the fact that, in the Middle East, the proportion of active sports men is still much higher than that of sports women. *Second*, measurements performed by a single investigator could be thought as a potential limitation, but we choose to be in line with the suggestion of Sernert et al. [36] and Ballantine et al. [37]; in evaluating the reproducibility of the KT-1000 arthrometer, the authors concluded that the KT-1000 evaluations and repeated measurements should be performed by the same examiner whenever possible to reduce the inter-personnel variability. *Third,* blinding, like in most of “surgical” studies, could not be performed; it would be obvious which knee was operated and which was not. *Fourth*, the effects of the arthroscopy itself, the swelling or the dressings on the knee laxity are not known and this constitutes a major limitation in our study. We hoped that in voiding the knee before taking the measurements and in using adhesive dressings, such effects could be, to some extent, reduced. *Fifth*, another limitation of this paper was the paucity in the literature of published studies dealing with the effect of meniscectomies on knee stability in the immediate post-operative period; it was not possible to compare and validate our findings. *Sixth*, our population consisted of young and active patients and it is possible that our results could not be generalized to other groups such as elderly subjects with degenerative meniscal tears.

## Conclusions

The present study included only 32 patients and conclusions should be drawn very carefully. The present data showed the value of anterior laxity measurements at MM of force under general anesthesia. Less than half of operated knees showed significant side-to-side difference values of ATT. Our values might reflect a temporarily increase of anterior laxity under specific conditions but whether a significant laxity remains in some knees, such changes may lead to higher cartilage loading and early osteoarthritis. Further research is needed in order to properly define the normal and abnormal value limits of the ATT between fully awake and anesthetized patients.

## Competing interests

The author declares that he has no competing interests.

## Pre-publication history

The pre-publication history for this paper can be accessed here:

http://www.biomedcentral.com/2052-1847/5/17/prepub
